# Dalbavancin is thermally stable at clinically relevant temperatures against methicillin-sensitive *Staphylococcus Aureus*

**DOI:** 10.5194/jbji-8-175-2023

**Published:** 2023-06-28

**Authors:** Aaron K. Hoyt, Patrick Lawler, Mathias Bostrom, Alberto V. Carli, Ashley E. Levack

**Affiliations:** 1 Department of Orthopaedic Surgery & Rehabilitation, Loyola University Medical Center, Maywood, IL 60153, USA; 2 Stritch School of Medicine, Loyola University, Maywood, IL 60153, USA; 3 Musculoskeletal Integrity Program, Hospital for Special Surgery, New York, NY 10021, USA; 4 Adult Reconstruction, Hospital for Special Surgery, New York, NY 10021, USA

## Abstract

**Introduction**: While the rate of orthopaedic infections has remained constant over the years, the burden on healthcare systems continues to rise with an aging population. Local antibiotic delivery via polymethyl methacrylate bone cement is a common adjunct in treating bone and joint infections. Dalbavancin is a novel lipoglycopeptide antibiotic in the same class as vancomycin that has shown efficacy against Gram-positive organisms when used systemically but has not been investigated as a local antibiotic. This study aims to identify whether dalbavancin is thermally stable at the temperatures expected during the polymerization of polymethyl methacrylate cement. **Methods**: Stock solutions of dalbavancin were prepared and heated using a polymerase chain reaction machine based upon previously defined models of curing temperatures in two clinically relevant models: a 10 mm polymethyl methacrylate bead and a polymethyl methacrylate articulating knee spacer model. Aliquots of heated dalbavancin were then transferred to be incubated at core body temperature (37 ${}^{\circ }$ C) and analyzed at various time points up to 28 d. The minimum inhibitory concentration at which 90 % of colonies were inhibited (MIC ${}_{90}$ ) for each heated sample was determined against methicillin-sensitive *Staphylococcus aureus* (American Type Culture Collection, ATCC, 0173K) using a standard microbroth dilution assay. **Results**: The average MIC ${}_{90}$ of dalbavancin was 1.63 $µg\;{\mathrm{mL}}^{-1}$ ±0.49 against 0173K *S. aureus*. There were no significant differences in the relative MIC ${}_{90}$ values after heating dalbavancin in either model compared to unheated control dalbavancin. **Conclusions**: Dalbavancin is thermally stable at the curing temperatures of polymethyl methacrylate cement and at human core body temperature over 28 d. Future in vitro and in vivo studies are warranted to further investigate the role of dalbavancin as a local antibiotic prior to its clinical use.

## 1 Introduction

Bone and joint infections remain a challenging clinical problem in orthopaedic surgery and often require prolonged treatment, including intravenous antibiotics and multiple surgeries; in some cases, these infections may even lead to amputation. The incidence of periprosthetic joint infection (PJI) is reported to range between 1 % and 2 % in primary joint replacement and up to 10 % in revision cases (Springer et al., 2017; Mortazavi et al., 2010; Dobson and Reed, 2020). Infection after internal fixation of closed fractures is reported to be approximately 1 %–5 % but increases to as high as 30 % in cases of open fractures (Gustilo and Anderson, 1976; Morgenstern et al., 2018; Papakostidis et al., 2011). These rates in orthopaedic trauma vary significantly when considering the location of the injury and the degree of soft-tissue injury, with increasing rates of infection with higher-grade Gustilo–Anderson open-fracture types (Papakostidis et al., 2011). Although the overall rate of bone infection appears low, because there is such a high prevalence of both total joint arthroplasty and fractures annually, surgical site infections pose a significant problem for orthopaedic surgeons.

The benefit of using local antibiotics is the high local concentration that can be achieved while maintaining low systemic concentrations, thereby lowering the risk of systemic side effects. Local antibiotic delivery using bone cement was first introduced in 1970 in the context of PJI in total hip arthroplasty with an overall first attempt treatment success rate of 77 % (Buchholz et al., 1981; Hake et al., 2015). Polymethyl methacrylate (PMMA) cement remains the most common delivery material for local antibiotic delivery. Since its introduction, antibiotic delivery using bone cement has evolved, resulting in approximately 90 % success in the treatment of PJI and between 70 % and 95 % success in the context of infection after fracture fixation or septic non-unions (Berend et al., 2013; Kuzyk et al., 2014; Lichstein et al., 2016). Although a large proportion of infections can successfully be eradicated, there remains a significant number of patients impacted by failure of these treatments, resulting in lower patient quality of life and a large economic burden on the healthcare system (Premkumar et al., 2021).

An increasing number of methicillin-resistant *Staphylococcus aureus* (MRSA) infections are failing treatment with traditional agents such as vancomycin, as many MRSA isolates are showing reduced susceptibility to this agent (McGuinness et al., 2017; Gardete and Tomasz, 2014; Appelbaum, 2007). Considering this, alternative agents to combat treatment-resistant infections are necessary. Dalbavancin is a lipoglycopeptide antibiotic that acts by interrupting cell wall synthesis, leading to bacterial death (Candiani et al., 1999). In vitro studies have demonstrated that dalbavancin has a higher potency against MRSA and other Gram-positive infections compared with vancomycin (Jones et al., 2005, 2001). Additionally, dalbavancin is in current use in the treatment of bone and joint infections as a systemic agent and has the advantage of being able to be dosed weekly as opposed to the daily dosing of vancomycin (Rappo et al., 2019; Almangour et al., 2019; Bouza et al., 2018; Dunne et al., 2015). It is of note that musculoskeletal infections are frequently biofilm-associated infections which often require higher antimicrobial concentrations to eradicate them, measured by the minimum biofilm eradication concentration (MBEC) (Zimmerli and Sendi, 2017). When comparing the MBEC to the traditional minimum inhibitory concentration (MIC), the MBEC of an antibiotic is often significantly higher; thus, higher concentrations of antibiotics may be necessary to combat these infections (Saginur et al., 2006). However, there are no clinically relevant performance standards for biofilm eradication at present (Malone et al., 2017).

While studies have shown that dalbavancin is a promising agent to treat MRSA infections that are less susceptible to vancomycin, there are few available data investigating the use of this agent as a local antibiotic in the context of bone and joint infections. For antibiotics to be considered for clinical use locally, it is important to define their efficacy through in vitro and in vivo studies. An important consideration is that the antibiotic agent used should be thermally stable at the temperatures produced during the exothermic cement polymerization reaction as well as at body temperature during the duration of expected antibiotic activity. For example, minocycline and meropenem do not retain their antimicrobial activity after heating in a 10 mm PMMA cement-bead model and subsequent incubation at human core body temperature (Levack et al., 2021). Additionally, an antibiotic must be stable over longer periods of time at human core body temperature when utilized in long-term carriers such as hydrogels or calcium sulfate.

This study aims to assess the thermal stability of dalbavancin through two clinically relevant models of PMMA cement: bone cement as a bead or as part of an articulating knee spacer. Further, by incubating at human core body temperature (37 ${}^{\circ }$ C) for 28 d, we also evaluated its long-term stability.

## 2 Methods

### 2.1 Antibiotic solution preparation

United States Pharmacopeia (USP) reference standards of dalbavancin (Dalvance^®^, Allergan USA, Inc., Madison, New Jersey) were obtained. Stock solutions of dalbavancin were prepared at concentrations of 1024 $µg\;{\mathrm{mL}}^{-1}$ with use of dimethyl sulfoxide (DMSO) solution, heated, and then stored at -80 ${}^{\circ }$ C in accordance with manufacturer guidelines about storage at this temperature and for ease of serial dilutions during testing.

### 2.2 Thermal stability testing

**Figure 1:**
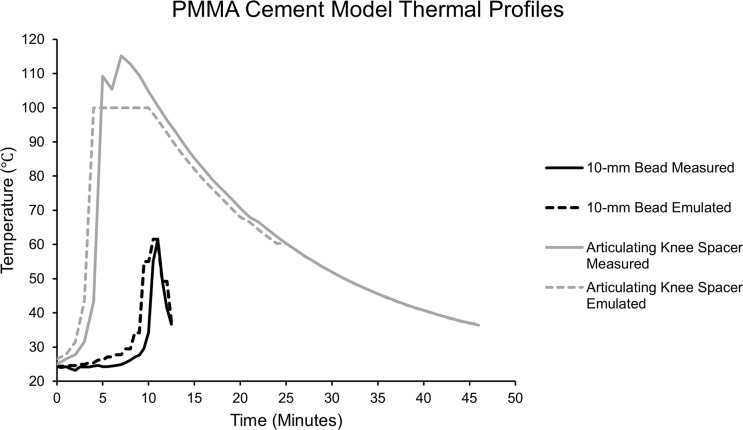
Simulated polymerization temperatures of a 10 mm PMMA bead and PMMA articulating knee spacer. The emulated temperature curves closely mimic the maximum temperatures recorded during the polymerization of each model.

Temperatures were measured during polymerization of two clinically relevant PMMA models: a 10 mm bead, the most common method of PMMA delivery in orthopaedic trauma, and an articulating knee spacer model (Levack et al., 2021). The cement was mixed according to manufacturer instructions and placed into the respective molds via manual mixing without vacuum (Simplex Bone Cement, Stryker, Kalamazoo, Michigan). Using a thermocouple, placed in the center of the bead (5 mm depth) or spacer (4 cm from the furthest surface and 1.5 cm from the closest surface) where maximum temperatures would be expected, the temperature in each model was measured at 30 s intervals until it reached human core body temperature (37 ${}^{\circ }$ C; Fig. 1). During 10 mm bead PMMA polymerization, a maximum temperature of 61.5 ${}^{\circ }$ C is recorded after 11 min, after which it rapidly cools over 2 min. The mold for the articulating knee spacer measured 8 cm × 5 cm × 3 cm (StageOne, Zimmer-Biomet, Warsaw, Indiana). During articulating knee spacer PMMA polymerization, the tibial component achieved higher temperatures and was thus used in this study as a more significant thermal challenge. In this model, a maximum temperature of 112.8 ${}^{\circ }$ C is recorded after 8 min, and it then gradually cools over 38 min. The emulated models closely mimic the measured thermal profile. These models reflect clinically relevant uses of local delivery via PMMA and generated temperatures that could challenge antibiotic stability.

Aqueous stock solutions of dalbavancin in aliquots of 2 mL were subjected to the simulated 25 min 10 mm bead thermal profile or simulated 44 min articulating knee spacer profile in a polymerase chain reaction (PCR) machine (vapo.protect Mastercycler; Eppendorf, Hamburg, Germany), followed by static incubation at human core temperature (37 ${}^{\circ }$ C). Samples of antibiotic solution were removed from incubation after intervals of 1 h, 24 h, 72 h, 7 d, 14 d, 21 d, and 28 d and were stored at -80 ${}^{\circ }$ C until further testing. The 0 h time point represents a sample of dalbavancin that was stored immediately after undergoing thermal profiling in a PCR machine. Dalbavancin elution from PMMA cement is modeled by incubation over the first 72 h. The latter time points were utilized to mimic long-term dalbavancin elution from stable carriers. The control group underwent no heating and was stored at -80 ${}^{\circ }$ C until testing.

### 2.3 Bacterial preparation

Methicillin sensitive *Staphylococcus aureus* (MSSA; American Type Culture Collection, ATCC, 0173K; Microbiologics, St. Cloud, Minnesota), which is derived from a clinical isolate parental strain of *S. aureus* (ATCC 0173K), was obtained. *S. aureus* was streaked on tryptic soy agar and incubated overnight. One colony was isolated into 5 mL of tryptic soy broth and placed in a shaking incubator for 24 h; this solution was then diluted by a factor of 50 and placed in a shaking incubator for 1.5 h. Bacteria were washed twice in phosphate-buffered saline (PBS) and resuspended in tryptic soy broth. Spectrophotometer absorbance (Spectramax Plus, Molecular Devices, Silicon Valley, California) was used to determine the optical density at 600 nm (OD600) of 0.490–0.510 A, indicative of log-phase growth. The bacterial solution was diluted to achieve a concentration of 10 ${}^{5}$ colony-forming units (CFUs). The OD600 solution was diluted and plated on tryptic soy agar to verify the concentration.

### 2.4 Microbiological assay

As previously described, a microbroth dilution assay was used to evaluate the minimum inhibitory concentration at which 90 % of the *S. aureus* isolates were inhibited (MIC ${}_{90})$ (Levack et al., 2022). Briefly, cation-adjusted Mueller–Hinton broth (CAMHB; Sigma-Aldrich, Darmstadt, Germany) was loaded into a 96-well plate, and serial dilutions of antibiotics were performed across the rows. Broth loading and serial dilutions were performed by an automated pipettor (OT-2; Opentrons, Brooklyn, New York) for increased accuracy and precision. Bacteria were added to each well at a concentration of $1\times 10^{5}$ CFUs. Plates were incubated at 37 ${}^{\circ }$ C for 24 h. Spectrophotometer absorbance was used to determine the cut-point concentration after which bacterial growth increases by beyond 10 %. A positive control column containing bacterial inoculation without antibiotics and a negative control column with neither bacterial inoculation nor antibiotics were used. Each heated sample was tested in quadruplicate and compared to an unheated control sample to determine the relative MIC of the heated and unheated samples.

### 2.5 Statistical analysis

MIC ${}_{90}$ values were reported as the average lowest concentration at which 90 % of bacterial growth was inhibited, and their standard deviations were also given. MIC values were compared to a control sample that was performed on each day of testing to create a relative MIC value. Relative MIC values at each heating time point were compared, using a one-way analysis of variance (ANOVA) with post hoc Dunnett’s t tests, to the unheated control. A p value of < 0.05 was considered significant. Given that only discrete MIC values can be obtained in a microbroth dilution assay, a standard deviation of zero represents excellent precision of the assay, whereas a standard deviation greater than zero represents an assay with one column of deviation. All statistical analyses were completed using SAS^®^ Studio (SAS Institute Inc., Cary, North Carolina, USA).

## 3 Results

### 3.1 Unheated control

The control group microbroth dilution assays of dalbavancin aqueous solutions produced a MIC ${}_{90}$ value of 1.63 $µg\;{\mathrm{mL}}^{-1}$ ±0.49 against 0173K *S. aureus*.

### 3.2 The 10 mm bead model

**Figure 2:**
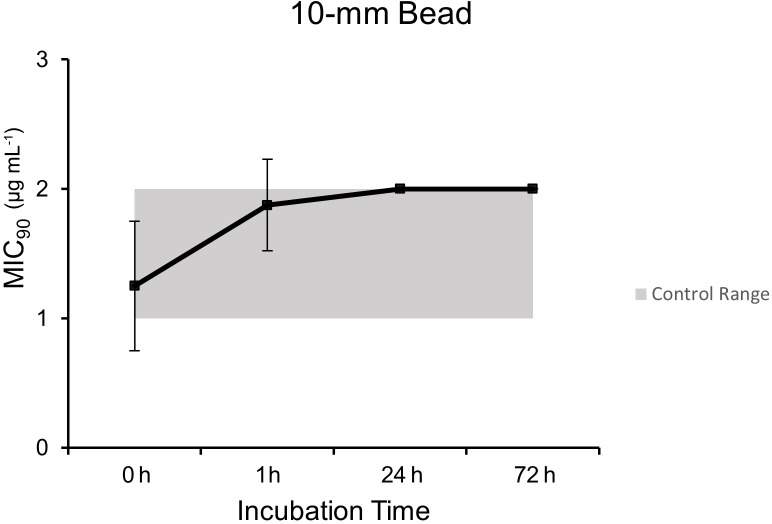
MIC ${}_{90}$ ( $µg\;{\mathrm{mL}}^{-1}$ ) over incubation times at human core temperature after heating under the simulated 10 mm PMMA bead model.

Microbroth dilution assays for samples exposed to the simulated thermal profile 10 mm PMMA bead model demonstrated comparable MIC ${}_{90}$ values to unheated control dalbavancin samples at each time point after post hoc testing ( p=0.019 ): 0 h (1.25 $µg\;{\mathrm{mL}}^{-1}$ ±0.5 ), 1 h (1.88 $µg\;{\mathrm{mL}}^{-1}$ ±0.35 ), 24 h (2.0 $µg\;{\mathrm{mL}}^{-1}$ ±0 ), and 72 h (2.0 $µg\;{\mathrm{mL}}^{-1}$ ±0 ) (Fig. 2).

### 3.3 Articulating knee spacer model

**Figure 3:**
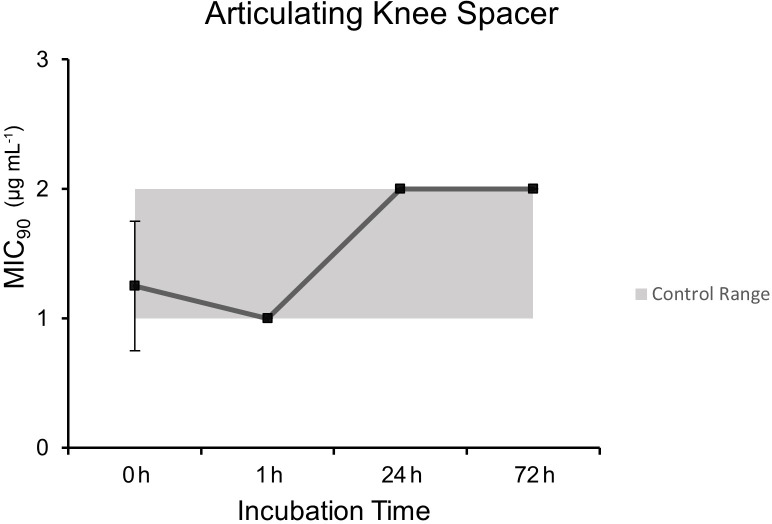
MIC ${}_{90}$ ( $µg\;{\mathrm{mL}}^{-1}$ ) over incubation times at human core temperature after heating under the simulated articulating knee spacer PMMA model.

Microbroth dilution assays performed after heating at curing temperatures of a PMMA articulating knee spacer model demonstrated comparable MIC ${}_{90}$ values to control unheated dalbavancin at each time point except for the 1 h time point ( p=0.0009 ): 0 h (1.25 $µg\;{\mathrm{mL}}^{-1}$ ±0.5 ), 1 h (1.0 $µg\;{\mathrm{mL}}^{-1}$ ±0 ), 24 h (2.0 $µg\;{\mathrm{mL}}^{-1}$ ±0 ), and 72 h (2.0 $µg\;{\mathrm{mL}}^{-1}$ ±0 ) (Fig. 3).

### 3.4 Long-term dalbavancin stability

**Figure 4:**
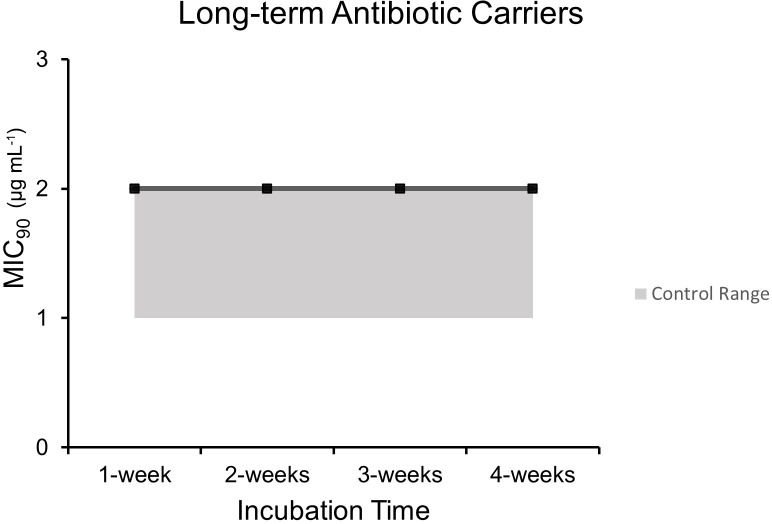
MIC ${}_{90}$ ( $µg\;{\mathrm{mL}}^{-1}$ ) over incubation times at human core temperature for up to 4 weeks to simulate dalbavancin's stability in long-term antibiotic carriers.

Microbroth dilution assays performed after incubation at human core body temperature over a period of 4 weeks to represent long-term antibiotic carriers such as hydrogels and calcium sulfate demonstrated comparable MIC ${}_{90}$ values to control unheated dalbavancin at each time point after post hoc testing ( p=0.0026 ): 1 week (2.0 $µg\;{\mathrm{mL}}^{-1}$ ±0 ), 2 weeks (2.0 $µg\;{\mathrm{mL}}^{-1}$ ±0 ), 3 weeks (2.0 $µg\;{\mathrm{mL}}^{-1}$ ±0 ), and 4 weeks (2.0 $µg\;{\mathrm{mL}}^{-1}$ ±0 ) (Fig. 4).

## 4 Discussion

As antibiotic resistance continues to be a growing problem in the management of orthopaedic infections, novel antibiotics that can be utilized locally will need to be identified. To the authors' knowledge, this is the first study evaluating dalbavancin, a novel antibiotic with great efficacy in orthopaedic infections when used systemically, in the context of local delivery via PMMA cement. While this study demonstrates that dalbavancin is thermally stable in the context of clinically relevant PMMA cement polymerization temperatures, this study is only one step in establishing dalbavancin as an efficacious local antibiotic in PMMA cement for the treatment of orthopaedic infections. Additionally, this study also provides evidence that dalbavancin is thermally stable over a 28 d period at human core body temperature; this demonstrates that it may be appropriate for study in multiple local carriers, including powders and hydrogels, as well as resorbable carriers, such as calcium sulfate, which have a slower antibiotic release.

These results lay the foundation for further evaluation of dalbavancin as a local antibiotic. It has been demonstrated that antibiotic elution is not necessarily improved with higher antibiotic loading in PMMA cement and that higher concentrations lead to decreased compressive mechanical properties (Slane et al., 2018). Future studies will need to evaluate the elution kinetics of dalbavancin in PMMA cement and other local antibiotic delivery materials to evaluate whether this antibiotic reaches concentrations over the course of treatment to adequately eradicate bacteria while also maintaining adequate compressive mechanical properties of the PMMA cement for its intended use. Additionally, future studies will be necessary to define the MIC of dalbavancin against multiple strains of clinically relevant bacteria in orthopaedic infections. Additionally, while dalbavancin has been shown to be safe when administered intravenously, including no reports of “red man syndrome” seen with vancomycin infusion, potential systemic toxicities include nausea, vomiting, diarrhea, pruritus, rash, and hypersensitivity reactions (Simonetti et al., 2021). While an advantage of local antibiotics is decreased systemic toxicities from lower systemic concentrations, future studies are necessary to identify any toxicities unique to the local delivery of dalbavancin as well as the appropriate concentrations to mitigate the risk of antibiotic resistance from overexposure.

A major limitation of this study is the absence of kinetic measurements of dalbavancin release from PMMA. Limitations of this study are related to the inherent limitations of in vitro study designs. This study does not mimic in vivo conditions, which can vary based on the local tissue environment. Additionally, this study only replicates two of the many clinical models of PMMA cement application in orthopaedic surgery. Although this study shows that dalbavancin is thermally stable over time at core body temperature as well as up to a peak temperature of 100 ${}^{\circ }$ C, there may also be slight alterations in the exact exothermic reaction that occurs when directly mixing the antibiotic with the PMMA cement during polymerization. Another limitation of this model is that the thermal testing was performed under open-air conditions during cement curing; thus, it is possible that higher temperatures would be achieved in the body. This model does not represent the highest temperatures that can be reached, as aqueous models are unable to be heated to the higher end of temperatures (120 ${}^{\circ }$ C) due to concerns regarding evaporation. This study also does not evaluate the elution kinetics of dalbavancin from PMMA cement, although we postulate that there would be similarities to other glycopeptide antibiotics such as vancomycin, which has been previously reported in the literature (Levack et al., 2021). While this study was not designed to assess the efficacy of dalbavancin against specific bacterial strains, it is notable that our average MIC ${}_{90}$ of 1.63 $µg\;{\mathrm{mL}}^{-1}$ against this specific strain of MSSA was higher than prior literature that has suggested an MIC ${}_{90}$ of around 0.125 $µg\;{\mathrm{mL}}^{-1}$ against other strains of MSSA, which is above the Clinical and Laboratory Standards Institute (CLSI) breakpoint of 0.25 $µg\;{\mathrm{mL}}^{-1}$ (Werth et al., 2021; Sader et al., 2021; Biedenbach et al., 2009, 2007; Goldstein et al., 2007; Jones et al., 2005; Gales et al., 2005). We caution the readers not to apply these data when assessing the clinical efficacy of dalbavancin against MSSA. Despite this, our comparison of relative MIC values demonstrates no comparative change in the MIC with and without the thermal challenge presented.

Overall, our study results demonstrate that dalbavancin is thermally stable during exothermic reactions in two clinically relevant models of PMMA cement use in orthopaedic surgery as well as at human core body temperature over a 28 d period. This study is foundational in establishing which local antibiotic carriers may be used for the local delivery of dalbavancin prior to its clinical use. Further studies are warranted to assess the clinical efficacy of dalbavancin against multiple strains of relevant Gram-positive organisms; the elution kinetics of dalbavancin from PMMA cement and other local delivery materials; the effect of dalbavancin on setting time, mechanical strength, and chemical interaction with PMMA; appropriate dosing of dalbavancin when used for local delivery; effectiveness against biofilm-associated infections; and in vivo models studying the effect of the local tissue environment on the efficacy of infection treatment prior to clinical use of dalbavancin as a local antibiotic.

## Data Availability

All data collected are presented graphically in the paper (Figs. 1–4).
